# Captive Bottlenose Dolphins Do Discriminate Human-Made Sounds Both Underwater and in the Air

**DOI:** 10.3389/fpsyg.2018.00055

**Published:** 2018-01-31

**Authors:** Alice Lima, Mélissa Sébilleau, Martin Boye, Candice Durand, Martine Hausberger, Alban Lemasson

**Affiliations:** ^1^Université de Rennes, Ethologie Animale et Humaine, UMR 6552, CNRS, Université de Caen Normandie, Paimpont, France; ^2^Département Scientifique et Pédagogique, Planète Sauvage, Port-Saint-Père, France; ^3^Cité Marine, Planète Sauvage, Port-Saint-Père, France; ^4^CNRS, Ethologie Animale et Humaine, UMR 6552, Université de Rennes, Université de Caen Normandie, Rennes, France

**Keywords:** individual-specific sound cue, individual identity, cetacean, auditory perception, bottlenose dolphins

## Abstract

Bottlenose dolphins (*Tursiops truncatus*) spontaneously emit individual acoustic signals that identify them to group members. We tested whether these cetaceans could learn artificial individual sound cues played underwater and whether they would generalize this learning to airborne sounds. Dolphins are thought to perceive only underwater sounds and their training depends largely on visual signals. We investigated the behavioral responses of seven dolphins in a group to learned human-made individual sound cues, played underwater and in the air. Dolphins recognized their own sound cue after hearing it underwater as they immediately moved toward the source, whereas when it was airborne they gazed more at the source of their own sound cue but did not approach it. We hypothesize that they perhaps detected modifications of the sound induced by air or were confused by the novelty of the situation, but nevertheless recognized they were being “targeted.” They did not respond when hearing another group member’s cue in either situation. This study provides further evidence that dolphins respond to individual-specific sounds and that these marine mammals possess some capacity for processing airborne acoustic signals.

## Introduction

Bottlenose dolphins are, with humans and a few species of birds, amongst the few species that have been shown to use learned individual-specific sound cues that use an individual’s identity to signal affiliation to group members (e.g., Henry et al., 2015; birds’ contact calls: Kondo and Watanabe, 2009 or songs: Hausberger et al., 1995; dolphins: Janik and Sayigh, 2013). Thus, they can copy the so-called signature whistles of others (Tyack, 1986) and these shared signature whistles constitute an affiliative signal that indicates strong social bonds (King et al., 2014). Other researchers have proposed that dolphins are able to “name” social partners by vocally copying one another and use this ability for spatial coordination (Janik and Sayigh, 2013; King et al., 2014). During evolution, the dolphin sensory world became primarily acoustic (Janik, 2013). In captivity, reports also show that they can learn to use acoustic signals consistently to report the presence or absence of particular objects, an ability shared with parrots (Pepperberg, 2007; King and Janik, 2013), and can label objects by copying artificial sounds (Richards et al., 1984). Miksis et al. (2002) found that dolphins can also incorporate features of artificial sounds made by humans into their own whistles. However, to our knowledge, the capacity of dolphins to learn artificial sound cues generated by humans has never been evaluated. The ability of animals to respond to individual sound cues (and not to respond to other group members’ sound cues) has been successfully experimentally tested in a limited number of species (monkeys – Masataka, 1992; pigs – Puppe et al., 2007). Previous studies have however clearly shown that individually trained captive dolphins learn gestural signals (Herman et al., 1984; Kuczaj et al., 2008). This modality was typically chosen because cetaceans are thought to perceive acoustic signals only underwater (Ketten, 2000).

Research on evoked auditory potentials or behavioral audiograms certainly emphasizes the adaptation of the cetacean hearing system to waterborne sounds (Erbe et al., 2016). In particular, their hearing system does not include an external auditory canal and their ossicular chain is stiff (Ridgway, 1988). Dolphins perceive sounds through their lower jaw, full of specialized fatty tissues that transmit sound directly to their middle and inner ears (Ketten, 2000). As a result, authors have questioned whether dolphins are able to perceive airborne sounds at normal intensity levels (Wartzok and Ketten, 1999; Erbe et al., 2016).

In two species of Delphinidae perception of airborne sounds has been tested (bottlenose dolphins: Babushina, 1979; a tucuxi: Liebschner et al., 2005). The results suggest that bottlenose dolphins and tucuxis are able to perceive certain airborne sounds, with hearing capacities in the air ranging from 1 to 110 kHz for the dolphin (Babushina, 1979; while different studies report hearing capacities underwater ranging from 0.075 to 180 kHz, review in Erbe et al., 2016) and from 2 to 31.5 kHz for the tucuxi (Liebschner et al., 2005; while underwater it ranges from 4 to 135 kHz; Sauerland and Dehnhardt, 1998). In these two studies, the animals were immobilized with the lower jaw out of water and a go/no go response paradigm was used to set the hearing thresholds. However sample sizes were small (two individuals in Babushina, 1979, and only one in Liebschner et al., 2005), while the subjects were restrained above water and not free to move or orient toward the sound source. Thus their ability to hear and to use airborne sounds remains unclear. Nevertheless, other indirect indications suggest that they may do so: captive bottlenose dolphins can mimic sounds broadcast in the air (Kremers et al., 2011), as do other odontocetes (e.g., belugas: Murayama et al., 2014). Moreover, training captive marine mammals by operant conditioning, with trainers using vocal signals, is common (Würsig, 2008). However, as trainers typically employ many different signals (e.g., gestural, postural, and vocal) simultaneously to give orders to the dolphins, it is difficult to know which signal is really effective.

In sum, it is known that (1) dolphins spontaneously use learned individual acoustic signals, (2) they are particularly sensitive to other sounds in captivity, (3) they can be conditioned using a set of signals of different types, (4) they have adapted to detect sounds underwater but there is some evidence of airborne sound perception. We thus carried out experiments to answer two questions: (1) Can dolphins respond underwater to learned sound cues artificially generated by humans? And if so, (2) Can dolphins generalize this learning to the airborne situation? Our predictions were that dolphins, like pigs, monkeys and dogs, would be able to respond appropriately to their own sound cues and ignore the sound cues allocated to other group members, even in the absence of any visual cues. We also hypothesized that dolphins would behave differently when hearing their own sound cue compared to other sound cues, when the same signals were transmitted through the air medium, thus generalizing from underwater learning to other conditions.

## Materials and Methods

### Captive Dolphins

A group of seven (three females and four males, aged 6–27 years old) captive bottlenose dolphins (*Tursiops truncatus*) born in different delphinariums and now all living in the “Cité Marine de Planète Sauvage” (Port-Saint-Père, France) delphinarium was studied (**Table 1**). They were housed as a group in four interconnected pools, containing approximately 8 million liters of water. These dolphins were fed fish (herring, capelin, mackerel, sprat, whiting, and squid) according to their individual needs, seven times a day. Two “free” meals were distributed at 9:00 to 17:00 and five others were distributed as a reward during training sessions or public presentations spread over the day. The training sessions lasted approximately 20 min and allowed trainers to condition the dolphins to perform certain actions for public performances as well as actions that facilitate medical care (taking temperature, blood sampling...).

**Table 1:** Instruments assigned to each individual and characteristics of the animals (M: male, F: female).

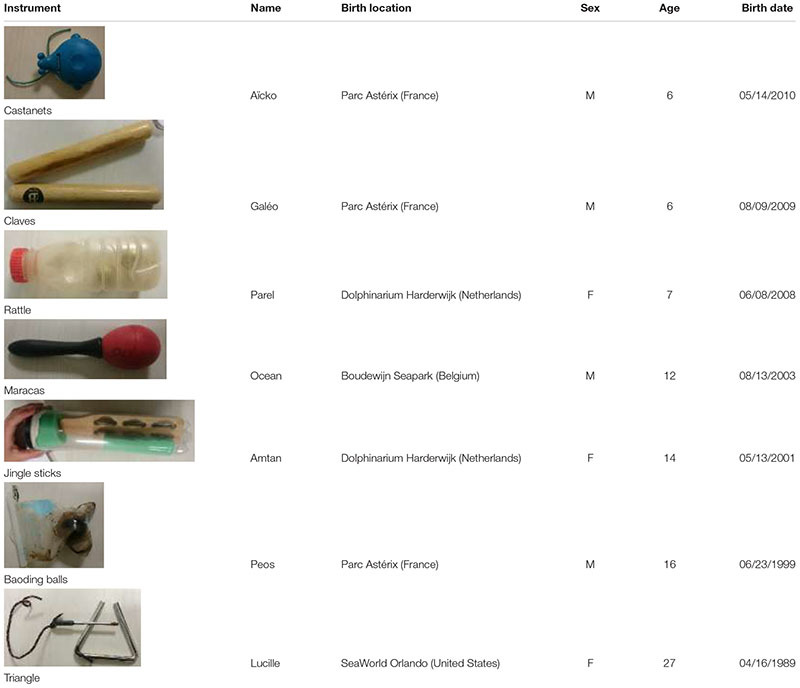

*Age in years. Photos: M. Sébilleau*.

All training, including habituation for medical or experimental procedures, was conducted by experienced caregivers using an operant conditioning technique based on positive reinforcement (mainly food, but also gelatine, ice cubes and enrichment items, like toys). During a regular training session, several trainers operate at the same time, each one dealing with one or more dolphins. Thus, dolphins are all busy executing different exercises, unless they prefer not to participate. They know the gestures telling them to come, to stay, and to leave, and during all sessions they are asked by gesture to make particular movements in exchange for reward.

Prior to our study, all these dolphins had been individually trained for at least 1 year to respond to a sound signal produced using a musical instrument, different for each animal (seven instruments were used, i.e., one per individual and always the same: castanets, claves, rattle, maracas, jingle sticks, Baoding balls, and a triangle; see **Table 1**). During weekly training sessions, a single trainer played an instrument underwater to “call” each dolphin. The instrument was immersed in the water at the edge of the pool, from varying locations, and played live; no recordings were used. Each dolphin was called, one after the other in a random order. The goal was to have the subject coming to the main trainer when hearing its designated individual sound. In the early stages of training, dolphins were first taught to approach and touch the instruments, being rewarded only when approaching and physically contacting the object. As learning improved, the main trainer played the instrument progressively farther away from the dolphin. In this exercise, the instrument was always first played twice, then a third time when the animal is halfway and finally twice when the animal is in front of the main trainer. If another trainer was working with the dolphin at the time of the instrument trial, he/she would give a “go” hand signal to allow it to go toward the sound source. Once the dolphin arrived at the sound source, it was given a food reward. A trial was considered successful when a dolphin moved toward the main trainer playing its designated instrument, and not toward the instrument assigned to another group member.

### Experimental Approach

Our first goal was to test under standardized conditions, whether dolphins successfully learned to respond to their individual sound source without the help of any visual cues (trainer gaze and gesture, instrument shape), without being influenced by the possible responses of the other group members, and without the need to play the instrument repeatedly to motivate the animal. We then tested their ability to generalize the training to airborne instrument playing. Responses to the sound signal to which they had been trained were thus evaluated first by playing the instruments underwater (Experiment 1: seven trials between 28th April and 4th May 2016, 1 per sound signal). Then, we recorded reactions to the same sound signal, but this time with instruments played in air (Experiment 2: seven trials between 6th and 16th May 2016, 1 per sound). For a given trial, only one sound was played and we performed only one or two trials per day. The order of the instruments tested was randomized for both experiments and only the main trainer was aware of the sequence.

Initially dolphins were tested in a group. Trials were always conducted in the same pool and with the same distance between the dolphins and the sound source (about 30 m). Dolphins were positioned with their backs to the main trainer (avoiding the possible use of echolocation), in the same position for all trials, and the sound source was hidden behind a plastic screen (to ensure that sound was the only cue for dolphins but also that trainers could not guess which dolphin would be called). Other trainers were instructed not to gesticulate or look at the subjects when the test started. The usual “go” gestural sign was thus never given by trainers for these experimental trials. The other trainers were not aware of the sound tested during a given trial and those familiar with the instrument allocated to each subject wore earplugs.

**FIGURE 1:**
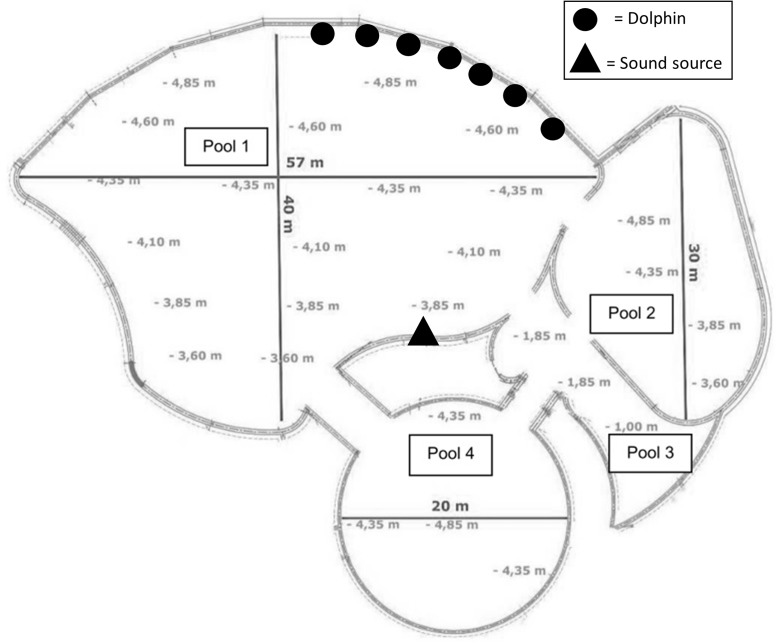
Schematic representation of the four pools at Planète Sauvage (adapted from Planète Sauvage). The symbols represent the position of individuals at the beginning of each test (“Dolphin,” random order of alignment) and the trainer operating the instruments (“sound source”).

At the beginning of a trial, several trainers (between 2 and 4) stayed near one side of pool 1 and thrashed the water in order to call the seven dolphins (**Figure 1**). Trainers placed all the dolphins in a “neutral” position, staying in place motionless, avoiding looking at the other dolphins, and allowing them to respond spontaneously (move or stay in place). When all the dolphins were in place with the right orientation, one of the seven instruments was played by the main trainer on the opposite side of the pool. The instrument was positioned approximately 30 cm under the surface of water (Experiment 1) or approximately 50 cm above the surface (Experiment 2).

All successful responses (a dolphin moves toward its designated sound, or it does not move when the sound is not its own specific sound cue) were rewarded when the dolphin arrived at the sound source or after 10 s when the dolphin did not move after hearing another sound cue. The behavior of the dolphins during the 10 s following playback was further analyzed: (1) we considered that a trial was successful when either the subject went toward the sound source when it was its own sound cue or when it did not move toward it when it was another dolphin sound cue; (2) we defined as a behavioral reaction any behavioral change; (3) we recorded temporal aspects such as latency of first reaction and duration of behaviors. Apart from locomotion, we also scored visual attention, estimated from head orientation as in Xitco et al. (2001).

Dolphin behavior was recorded during the trials with three cameras: one underwater [Sport digital compact camera PNJ Cam AEE S71 High Definition (HD)] below the sound source and two others (Sony HDR-XR155E) placed above the water, one filming the area where the main trainer played the instruments and the other focused on the dolphins’ starting position. We also used a broadband system consisting of a C54XRS (-185 dB, re 1V/μPa, 0.06 to 203 kHz) hydrophone connected to a TASCAM DR-680 recorder (sampling rate 192 kHz 24 bit) to confirm that instruments, when played in the air, could not be heard underwater by dolphins at the test position (30 m away).

### Ethics Statement

The experiments described in this paper were carried out in accordance with the current laws of the country in which they were performed. They complied with the current French laws (Centre National de la Recherche Scientifique) related to animal experimentation and were in accordance with the European directive 86/609/CEE. The research was approved by the “Direction Départementale de la Protection des Populations” committee of Loire-Atlantique prefecture. No further permit was needed as only behavioral observations were performed. Animal husbandry and veterinary care were under management of Planète Sauvage, from whom informed consent had been obtained, as this study involved animals from a private animal park (not laboratory animals) with whom informed consent has been granted.

### Data and Statistical Analyses

The behavioral data for the seven dolphins were analyzed using the focal sampling method (Altmann, 1974) during the 10 s of each test following the playing of an instrument. Movements and gazes as well as their targets were recorded (**Table 2**). Responses were classified as successful or failed (successful when the dolphin reached the sound source within 10 s after hearing its own sound cue or when not moving after hearing another dolphin’s sound cue; failed when it did not respond to its own sound cue or responded to another dolphin’s sound cue. We analyzed the first reaction (i.e., first change of behavior) and its occurrence, duration and latency.

**Table 2:** Terminology of behaviors observed during the experiment.

Behavior		Description
Movement	Source	Dolphin moves to the sound source.
	Trainer	Dolphin moves to another trainer.
	Other	Dolphin moves in a direction other than that of the sound source or a trainer.
Gaze (Starting position - Dolphin on the edge of the pool)	Source	Dolphin looks in the direction of the sound source.


	Trainer	Dolphin looks in the direction of one of the trainers.
	Conspecific	Dolphin looks in the direction of a conspecific.
	Other	Dolphin looks in a direction that does not correspond to trainers, sound source or conspecific.

A binomial test compared movement toward source between own and other sounds. Chi-square tests compared the numbers of dolphins performing each behavior in each situation. In order to compare changes of gaze direction, duration and latency in relation to the type of sound (i.e., own or other), we used Wilcoxon tests. Comparisons between reactions to own and other sound cues were computed using reactions to the broadcast of own sound cue to the median of this subject’s reactions to the six playbacks of the other individual sound cues. We performed all statistical analyses with the software R 3.2.2. (R Core Team, 2014).

### Data Availability

The datasets generated during and/or analyzed during the current study are available from the corresponding author on reasonable request.

## Results

### Experiment 1: Underwater Broadcast Conditions

**FIGURE 2:**
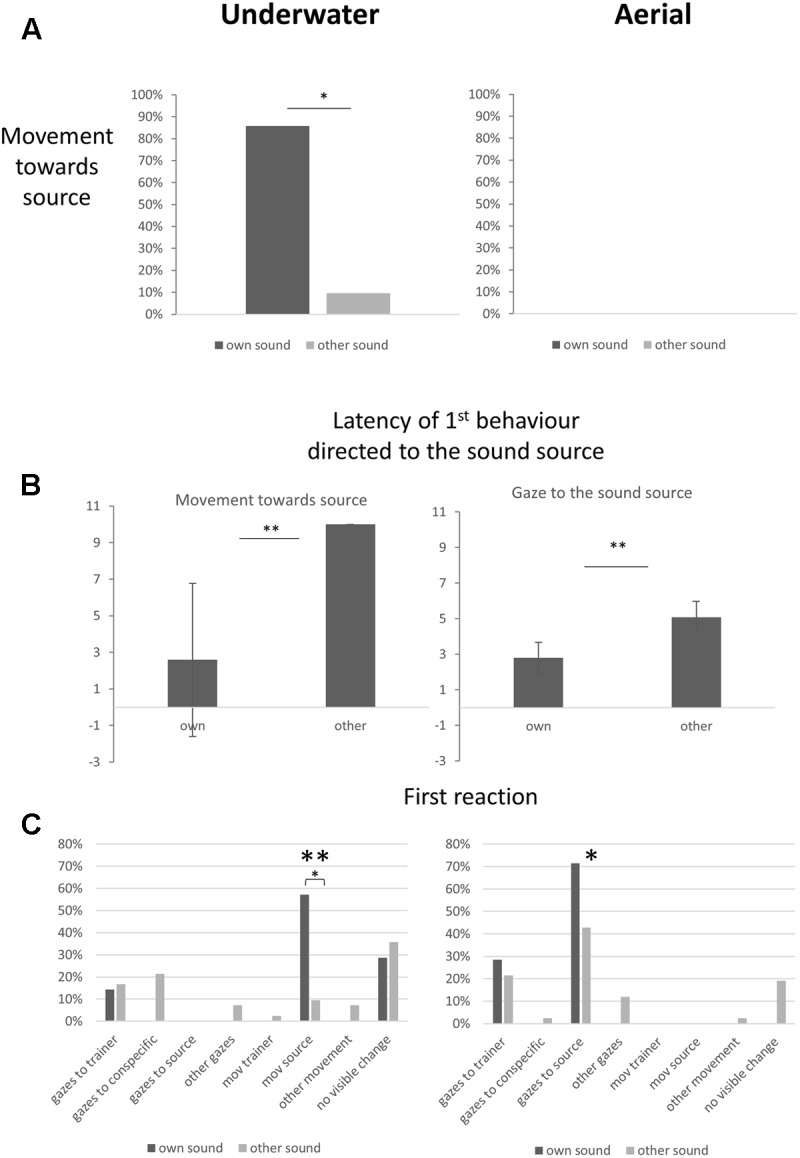
Dolphin reactions to their own individual sound cue and to other sound cues broadcast underwater (left) and in the air (right). **(A)** Movements of dolphins toward the sound source (percentage of subjects; binomial test on real numbers, ^∗^*P* < 0.05). **(B)** Latencies of first reactions to the sound: movement when the sound was underwater and gaze when it was airborne; mean ± standard error (Wilcoxon test, medians of latencies per subject for other individual-specific sound cues versus own sound cue, ^∗∗^*P* < 0.01). **(C)** Dolphins’ first reactions to a broadcast (percentage of subjects). One behavior predominated in each situation: movement toward the source of the underwater sound, gaze toward the source of the airborne sound (chi-square test performed on real numbers, ^∗^*P* < 0.05, ^∗∗^*P* < 0.01).

In the first experiment, dolphins were exposed to sounds emanating from one of seven instruments played underwater. Each dolphin was trained to respond to the sound of only one instrument. Responses to each instrument were recorded, as were latencies to approach. All the dolphins except one moved toward the main trainer located near the sound source when their individual instrument was played (six out of seven trials, binomial test, *P* < 0.05) (**Figure 2**). They did not move in 90.5% of the trials when the individual sound cue of another dolphin was broadcast. Overall, the dolphins were successful, 89.8% responses were appropriate (Sign test, *P* < 0.01). Failures included one (1/7) subject (Ocean, see **Table 1**) that did not react when its individual sound cue was broadcast and four subjects that went once (out of seven trials each) toward a sound cue that was not their own. Moreover latencies to move toward the source were shorter when they heard their own sound cue (**Figure 2**). The target animal did not gaze before departing. Throughout the session the other dolphins looked either at their trainer (mean: 0.97 ± 0.13 gazes), at another feature of the environment (mean: 0.53 ± 0.12), at a conspecific (mean: 0.46 ± 0.10) or at the sound source (mean: 0.03 ± 0.02). Number of gazes directed to a conspecific (*V* = 89, *P* = 0.45), a trainer (*V* = 101, *P* = 0.15) or other features (*V* = 100, *P* = 0.11) did not differ significantly in relation to the sound broadcast.

### Experiment 2: Aerial Broadcast Conditions (**Figure 2**)

In the second experiment, dolphins were exposed to sounds emanating from the same seven instruments, but this time played in the air. Dolphins had only been trained to respond to these sounds when played underwater. Responses to each instrument were recorded, as were duration and latencies of gazing at the sound source, at a trainer or at other objects of the environment. None of the dolphins moved toward the sound source during the tests (**Figure 2**). In 91% of the trials, dolphins had their heads (lower jaws) out of the water at the time of sound emission. Behavioral responses indicated that the dolphins not only heard the sounds but even discriminated between them: (1) latencies of first gaze toward the source were shorter when the sound broadcast was their own sound cue (**Figure 2**); (2) the type of the sound broadcast did not influence the number of gazes directed toward the source (*V* = 13.5, *P* > 0.05), but it influenced the total time spent looking at the source (*V* = 28, *P* < 0.05): time spent looking at the source was 5.09 (± 0.3) seconds when the sound broadcast was the subject’s own sound cue, but only 2.40 (± 0.6) seconds when it was another dolphin’s sound cue; (3) the dolphins’ first reaction following the stimulus broadcast was generally to look at the sound source (chi-square test, *n* = 7, DF = 6, *P* < 0.01) (**Figure 2**). Number of gazes directed toward a conspecific (*V* = 133.5, *P* = 0.37), a trainer (*V* = 130.5, *P* = 0.62) or other features of the environment (*V* = 126, *P* = 0.52) did not differ significantly in relation to the sound broadcast.

### Comparison between Underwater and Aerial Conditions

Number and duration of gazes toward the sound source were significantly higher when the broadcast was aerial (Wilcoxon Tests: *V* = 354, *P* < 0.01; *V* = 343, *P* < 0.01, respectively). As a consequence, duration of gazes toward conspecifics was significantly shorter in the aerial condition than in the underwater condition (0.46 ± 0.1 underwater and 0.08 ± 0.0 s in the air, *V* = 1098, *P* < 0.01). Conversely, movements toward the sound source were restricted to the underwater condition (**Figure 2**).

Number (*V* = 88.5, *P* = 0.21) and duration (*V* = 101, *P* = 0.27) of gazes toward other targets were not influenced by the type of broadcast.

## Discussion

This study provides the first evidence that bottlenose dolphins can recognize a human-made sound cue played underwater, even when transposed to the aerial environment. Prior to these experiments, dolphins had been trained individually with the possible help of visual cues. Here, dolphins were tested while in a group with exclusively auditory stimuli, and we confirmed not only that the target subject responded to its specific human-made sound cue but also that it did not move when another group member was “called.”

In the first experiment, the dolphins performed the trained response (moved toward the source). Operant conditioning is widely used for the management and training of captive dolphins. Daily training and public performances are based on teaching the animals gestures associated with specific behaviors. Dolphins are able to associate a human movement with a specific action, or a specific part of their body, and can respond to orders combining these different elements thanks to their understanding of simple syntax rules (Herman, 2002; Herman et al., 1984). Furthermore, they can incorporate features of artificial sounds made by humans into their whistles (Miksis et al., 2002) or use novel sounds to refer to objects (Richards et al., 1984). They are also said to be self-aware, notably because they recognize their bodies in a mirror (Reiss and Marino, 2001). Their high-level cognitive abilities are further shown by imitation of computer-generated sounds, and of postural or motor behaviors of non-cetacean species and dolphin tank mates (Richards et al., 1984; Reiss and McCowan, 1993; Herman, 2002; King and Janik, 2013). They signal individual identity through signature whistles that they can share with other dolphins through affiliative copying (Harley, 2008; King et al., 2013), which implies labeling, a skill shared with humans and some bird species (Hausberger et al., 1995; Wanker et al., 2005; Pepperberg, 2007). Our findings suggest that dolphins can associate sound cues with individual identities and we believe that this contributes to the debate regarding the potential existence of a concept of self-identity in dolphins. Responding to its own “label” and not to that of other group members is definitely not restricted to humans (Masataka, 1992; Puppe et al., 2007). Further experiments could test their ability to associate a given sound cue to the image of the appropriate group member, for example.

In the second experiment, dolphins responded differently when the sounds were played in the air: they did not move toward the sound source, so did not generalize the training *per se*, but clearly discriminated between their own and other individual sound cues. Thus, they gazed more often and for longer toward the sound source and reacted faster when their own sound cue was being broadcast. The aerial hearing sensitivity of dolphins has been debated, and some authors doubt that cetaceans are really able to hear sounds emitted in the air (Ketten, 2000). Marine mammal ears are adapted to aquatic life (Breathnach et al., 1988). The absence of a functional ear canal similar to that of terrestrial mammals makes them less sensitive to sounds in the air and is probably one of the factors that accelerated the specialization of their inner and middle ear for perception of sounds underwater (Hemilä et al., 2010).

Nonetheless, most auditory studies have focused on reception of waterborne sounds (review in Erbe et al., 2016) and only three studies (bottlenose dolphins: Babushina, 1979; harbor porpoises: Kastelein et al., 1997 and tucuxis: Liebschner et al., 2005) investigated sensitivity to airborne sounds. These authors suggested that dolphins are able to perceive acoustic stimuli broadcast in air. Kremers et al. (2011) and Murayama et al. (2014) supported this by reporting imitation by odontocetes of aerial sounds from the environment (airborne playback of whale sounds or human speech). The fact that our dolphins reacted faster when the sound signal broadcast was their own sound cue than when it was that of a conspecific shows that the dolphins were able to perceive and recognize sound signals diffused in the air. The difference in reactions to underwater and airborne acoustic stimuli may be due to either poor hearing (Liebschner et al., 2005), inability to generalize the learned response, or more likely to a “surprise” effect like that observed when unexpected sounds are heard (e.g., Lemasson et al., 2005) or in the context of an expectancy violation paradigm (e.g., Kastelein et al., 1997). They gazed more at the sound source when the instrument was played in the aerial condition, which could be an indication that they were trying to understand the demand, as shown in other studies when humans behaved unexpectedly or differently (Xitco et al., 2001). Thus, horses increased their monitoring behavior after hearing a familiar order given by an unknown person (Sankey et al., 2011). Dolphins look more at their trainer when their performance is inconsistent during a familiar task (Xitco et al., 2004). Dolphins could use cues based on human movements, as they are trained to be very attentive to gestures during training sessions (Tomonaga et al., 2015). The fact that dolphins possibly search for clues given by human postures could explain the multiple gazes toward the source.

## Conclusion

This study shows evidence that bottlenose dolphins are able to respond to individual sound cues produced by humans, even when sounds are emitted in the air. This evidence contributes to our knowledge of the cognitive capacities of this species and the extension of its hearing capabilities. Further studies could test if dolphins can associate these sound cues with individual identities.

## Author Contributions

ALi, MS, MB, MH, and ALe designed the research project; ALi, CD, and MS performed the experiments; ALi, MS, MH, and ALe contributed to data analyses; and ALi, MS, MH, and ALe wrote the paper.

## Conflict of Interest Statement

The authors declare that the research was conducted in the absence of any commercial or financial relationships that could be construed as a potential conflict of interest.
